# Real-world experience with belimumab in lupus nephritis: insights from the Spanish cohort

**DOI:** 10.3389/fimmu.2026.1897450

**Published:** 2026-08-25

**Authors:** Ana Huerta, Paula López-Sánchez, Eduardo Martínez, Luis F. Quintana, Adoración Martín-Gómez, Vanessa Lopes, Cristina Rabasco, Jorge Alberto Rodriguez, Celia Rodriguez, Alba Temprado, Manuel Lopez-Mendoza, Francisco Jose Borrego, Sara Afonso, Antolina Rodriguez, Loreto Fernandez, Maria Gago, Maria del Carmen Prados, Olga Gracia, Milagros Sierra, Marina Sánchez-Agesta, Javier Carbayo, Fernando Hadad, Ana Romera, Roger Cox, Jose Maria Mora, Begoña Rivas, Juan Fernandez-Solis, Francisco Gomez-Preciado, Maria Teresa Mora, Eva Marquez, Mar Espino, Carmen Larramendi, Mireia Lopez-Corbeto, Adriana Devolder-Nicolau, Adriana Puente-Garcia, Amir Shabaka, Sara Gonzalez, Carmen Martin, Carmen Mon, Ana Tato, Daniel Villa, Maria Galindo, Lucia Martin-Testillano, Jose Portolés, Enrique Morales

**Affiliations:** 1 Department of Nephrology, Hospital Puerta de Hierro Majadahonda, Madrid, Spain; 2 Instituto Investigación Puerta de Hierro-Segovia Arana, IDIPHISA, Madrid, Spain; 3 Department of Nephrology, Hospital Clinic, Universidad de Barcelona, Instituto de Investigaciones Biomédicas August Pi i Sunyer (IDIBAPS), Barcelona, Spain; 4 Department of Nephrology, Hospital Universitario Poniente, Almería, Spain; 5 Department of Nephrology, Hospital Universitario Ramon y Cajal, Madrid, Spain; 6 Department of Nephrology, Hospital Universitario Reina Sofia, Cordoba, Spain; 7 Department of Nephrology, Hospital Universitario de Badajoz, Badajoz, Spain; 8 Department of Nephrology, Hospital Universitario de Salamanca, Salamanca, Spain; 9 Department of Nephrology, Hospital Universitario Virgen del Rocio, Sevilla, Spain; 10 Department of Nephrology, Hospital Universitario de Jaen, Jaen, Spain; 11 Department of Nephrology, Hospital Universitario Insular de Gran Canaria, Gran Canaria, Spain; 12 Department of Nephrology, Hospital Universitario Clinico San Carlos, Madrid, Spain; 13 Department of Nephrology, Hospital Universitario de Navarra, Navarra, Spain; 14 Department of Nephrology, Hospital Universitario Central de Asturias, Asturias, Spain; 15 Department of Nephrology, Hospital Universitario Torrecardenas, Almería, Spain; 16 Department of Nephrology, Hospital Universitario Miguel Servet, Zaragoza, Spain; 17 Department of Nephrology, Hospital Universitario San Pedro, La Rioja, Spain; 18 Department of Nephrology, Hospital Universitario Puerto Real, Cadiz, Spain; 19 Department of Nephrology, Hospital Universitario Gregorio Marañon, Madrid, Spain; 20 Department of Nephrology, Hospital Clinico Universitario Virgen de la Arrixaca, Murcia, Spain; 21 Department of Nephrology, Hospital General Universitario de Ciudad Real, Ciudad Real, Spain; 22 Department of Nephrology, Clínica Universitaria de Navarra, Navarra, Spain; 23 Department of Nephrology, Hospital Universitario La Paz, Madrid, Spain; 24 Department of Nephrology, Hospital del Mar, Barcelona, Spain; 25 Department of Nephrology, Hospital Universitario de Bellvitge, Barcelona, Spain; 26 Department of Nephrology, Hospital Universitario Juan Ramon Jimenez, Huelva, Spain; 27 Department of Pediatric Nephrology, Hospital Universitario 12 de Octubre, Madrid, Spain; 28 Department of Pediatric Nephrology, Hospital Universitario Vall d’Hebron, Barcelona, Spain; 29 Unit of Pediatric Rheumatology, Hospital Universitario Vall d’Hebron, Barcelona, Spain; 30 Department of Pediatric Nephrology, Hospital de Sant Pau, Barcelona, Spain; 31 Department of Pediatric Nephrology, Hospital Universitario de Fuenlabrada, Madrid, Spain; 32 Department of Nephrology, Hospital Universitario Germans Trias, Barcelona, Spain; 33 Department of Nephrology, Hospital General de Segovia, Segovia, Spain; 34 Department of Nephrology, Hospital Universitario Severo Ochoa, Madrid, Spain; 35 Department of Nephrology, Hospital Universitario Fundacion Alcorcon, Madrid, Spain; 36 Department of Rheumatology, Hospital Universitario 12 de Octubre, Madrid, Spain; 37 Department of Nephrology, Hospital Universitario 12 de Octubre, Madrid, Spain

**Keywords:** anti-dsDNA reduction, belimumab, early response, lupus nephritis, maintenance

## Abstract

**Objectives:**

Belimumab has emerged as a therapeutic option for lupus nephritis (LN); however, real-world evidence on its effectiveness remains limited. This study aimed to evaluate the effectiveness and safety of belimumab and to identify renal and immunological predictors of response in Spanish patients with LN.

**Methods:**

We conducted a retrospective, multicenter study including patients with biopsy-confirmed LN treated with belimumab between 2012 and 2024. Outcomes included primary efficacy renal response (PERR), complete renal response (CRR), changes in proteinuria, estimated glomerular filtration rate (eGFR), urinary sediment, histological parameters, and immunological serologic markers, including anti–double-stranded DNA antibodies (anti-dsDNA), complement components C3 and C4, and erythrocyte sedimentation rate(ESR). Renal flares were defined according to the BLISS-LN criteria. Data on disease activity (SLEDAI-2K), adverse events, and concomitant immunosuppressive therapies were also collected.

**Results:**

The study included 202 patients, predominantly women (87.5%), with mainly proliferative LN (79.7%). At 6, 12, and 24 months, PERR was achieved in 70.7%, 71.1%, and 72.7% of patients, respectively, while CRR rates were 52.4%, 47.6%, and 49.1%. These outcomes were consistent with those reported in the BLISS-LN and BeRLiSS-LN trials. Early initiation of belimumab was associated with greater improvement in renal and immunological serologic markers. A ≥50% reduction in anti-dsDNA levels at 12 months was associated with higher odds of achieving PERR (OR 3.91, 95%CI 1.00–15.30; *p* = 0.050). In a small subgroup undergoing repeat biopsy, histological activity index improved after treatment initiation. SLEDAI-2K score significantly decreased (p<0.001), accompanied by a low rate of renal flares. Prednisone and mycophenolate mofetil (MMF) doses decreased significantly during follow-up. Belimumab was well tolerated, with few discontinuations due to adverse events.

**Conclusions:**

Belimumab use was associated with maintenance or improvement of renal response in a real-world cohort of patients with LN. The apparent benefits of early initiation, together with the observed reductions in corticosteroid and MMF doses, the favorable safety profile, and the low rate of renal flares, suggest that belimumab may be an effective option both in early disease and for long-term management. Furthermore, a ≥50% reduction in anti-dsDNA antibody titers may represent a useful prognostic biomarker of renal response in patients with predominantly proliferative LN treated with belimumab.

## Introduction

Lupus nephritis (LN) is a common complication in Systemic Lupus Erythematosus (SLE), occurring in more than half of SLE patients throughout the course of their disease. Unfortunately, it is a severe complication that negatively impacts the prognosis of these patients (1, 2). In recent years, new therapeutic tools have become available, showing promising results for these patients (3–5*)*.

Belimumab is a human IgG1λ monoclonal antibody that specifically binds to the soluble form of the human B-lymphocyte stimulator (BLyS) protein, also known as BAFF and TNFSF13B. It blocks the binding of soluble BLyS, a B-cell survival factor, to its receptors on B-cells, thereby inhibiting B-cell survival, including autoreactive B-cells, and reducing the differentiation of B-cells into immunoglobulin-producing plasma cells. BLyS levels are elevated in patients with SLE and other autoimmune diseases. A correlation has been observed between BLyS levels and disease activity in SLE (6, 7*)*. Belimumab has demonstrated both efficacy and safety in clinical trials involving patients with SLE, including those with LN *(*3, 8–14*)*, and currently, clinical practice guidelines support the early use of belimumab as an add-on component of initial combination therapy for active lupus nephritis *(*15–17*)*.

However, scientific evidence regarding the effect of belimumab in real-world patients with LN remains limited, and most of the available data come from Asian populations *(*18–22*)*. Here, we present a large cohort of patients from Spain evaluating the effectiveness, safety, and clinical benefits of belimumab in patients with LN. In addition, we describe renal and immunological predictors of response identified in our cohort of LN patients treated with belimumab.

## Materials and methods

### Patient population

We collected data from patients with LN treated with belimumab in Spain between 2012 and 2024. Inclusion criteria were: (a) fulfilment of the 2019 EULAR/ACR classification criteria for SLE *(*23) (b) a biopsy-confirmed diagnosis of LN class III, IV, V, a combination of class III or IV with V, or lupus podocytopathy; and (c) treatment with belimumab. Exclusion criteria were: (a) evidence of an alternative nephropathy on renal biopsy; and (b) follow-up of less than 6 months. The study was approved by the Institutional Review Board of Puerta de Hierro University Hospital, Madrid (ref: 67/135343.9/23).

### Data collection

Clinical and laboratory data were collected retrospectively from medical records. Demographic variables included age, sex, race or ethnicity, and comorbidities (hypertension and diabetes) at presentation. Initiation of belimumab was defined as baseline. Laboratory variables included serum creatinine, estimated glomerular filtration rate (eGFR) calculated with the Chronic Kidney Disease Epidemiology Collaboration (CKD-EPI) equation, serum albumin, and urinary protein excretion (24-hour urine protein; the urinary protein-to-creatinine ratio was used when 24-hour collections were unavailable), as well as urinary sediment, anti–double-stranded DNA (anti-dsDNA) antibodies, serum complement components C3 and C4, and erythrocyte sedimentation rate (ESR). Treatment data included use of antimalarials, renin–angiotensin–aldosterone system (RAAS) blockers, sodium–glucose cotransporter 2 inhibitors (SGLT2i), and other immunosuppressive agents. Mycophenolic acid doses were converted to equivalent mycophenolate mofetil (MMF) doses. Clinical disease activity was assessed with the SLE Disease Activity Index 2000 (SLEDAI-2K). Adverse events related to belimumab were also recorded. Histological variables included LN class, activity and chronicity indices *(*24*)* and the percentage of global sclerosis, interstitial fibrosis, and crescents.

### Outcomes and definitions

The primary outcome was renal response to belimumab treatment, defined according to the BLISS-LN study criteria. “Primary efficacy renal response” (PERR) was defined as proteinuria ≤0.7 g/24 h, an eGFR ≥60 mL/min/1.73 m² or no more than a 20% decrease from baseline, and no use of rescue therapy. “Complete renal response” (CRR) was defined as proteinuria <0.5 g/24 h, an eGFR ≥90 mL/min/1.73 m² or no more than a 10% decrease from baseline, and no use of rescue therapy *(*3; **Supplementary Methods S1**). To enable direct comparison with the BeRLiSS-LN study, we applied the same definitions employed in that study *(*18; **Supplementary Methods S1**).

Secondary outcomes included changes in laboratory parameters (eGFR, proteinuria, hematuria, serum C3 and C4, and anti-dsDNA antibodies), SLEDAI-2K score, doses of concomitant immunosuppressive therapy, histological parameters, LN flares, and adverse events. All outcomes were assessed at 6, 12, and 24 months. LN flares were defined according to the *post hoc* analysis of the BLISS-LN trial *(*12*;* **Supplementary Methods S1**) and were assessed from week 24 onward in patients who had achieved PERR at week 24. “Early initiation” was defined as starting belimumab within 1 month of histological diagnosis. This time threshold was selected in line with previous time-based analyses in other immune-mediated diseases, particularly ANCA-associated vasculitis with renal involvement *(*25*).*

### Statistical analysis

Data are presented as mean ± standard deviation (SD) or median and interquartile range [IQR], depending on the distribution of the variables. We estimated the decrease in immunological parameters (C3 and anti-dsDNA) during the 12 months following belimumab initiation, categorizing the relative reduction as >50%, 25–50%, or <25%. Comparisons between groups were performed using the Student’s t-test, Mann–Whitney U test, or Chi-squared test, as appropriate. For paired pre–post comparisons, the paired Student’s t-test was used. Time to achieve either a renal response or complete response was estimated using the Kaplan–Meier method in the population without a response at baseline.

A logistic analysis was conducted to identify risk factors associated with PERR at 24 months, with results expressed as odds ratios (OR) and 95% confidence intervals (95% CI). Variables with a *p*- value < 0.10 in the univariable analysis were considered eligible for inclusion in the multivariable model. In addition, predictors judged to be clinically relevant *a priori* were included regardless of their univariable statistical significance, to minimize potential residual confounding and to ensure that the final model reflected established clinical knowledge. Model fit was evaluated using McFadden’s pseudo-R². No imputation was performed for missing data; analyses were conducted using pairwise deletion, including all available data for each analysis. A *p* value < 0.05 was considered statistically significant. Statistical analyses were performed using Stata (StataCorp. 2019. Stata Statistical Software: Release 16. College Station, TX: StataCorp LLC).

## Results

Data were collected from 237 patients across 35 hospitals in Spain, with 35 patients excluded from analysis (**Supplementary Figure 1**).

### Baseline data

A total of 202 patients were included in the analysis (**Table 1**). The cohort was predominantly female (87.5%), with 69.3% Caucasian and 22.8% Hispanic/Latino. All patients underwent renal biopsy, with 79.7% exhibiting proliferative LN (class III or IV) and 10.4% class V. The median time from SLE diagnosis to renal biopsy was 2 years [IQR 0–7], and the median interval from biopsy to belimumab initiation was 1.2 years [IQR 0.07–6.9]. Of the 202 patients included, only 59 completed 24 months of follow-up. The remaining patients had not reached this timepoint by the end of the study follow-up period or discontinued treatment before completing 24 months.

**Table 1:** Baseline data.

N	202
Age (y)	36.9 (13.2)
Male (%)	12.5
Ethnicity (%)	
Caucasian	69.3
African American	4
Asian	4
Hispanic/Latino	22.8
**Hypertension (%)**	**34.2**
**Diabetes Mellitus (%)**	**2.5**
**Patients with previous relapses (%)**	**52.5**
**Follow-up (y)**	**1.1 [0.7–2.2]**
Histology	
Classes III or IV (%)	79.7
Class V (%)	10.4
Mixed class (%)	8.9
Podocytopathy (%)	1
Activity index	7.1 (4.6)
Chronicity index	1.9 (2.5)
Globally sclerosed glomeruli (%)	5.3 (13.6)
Crescents (%)	4.5 (13.5)
Interstitial fibrosis (%)	9.4 (12.3)
Belimumab initiation	
Subcutaneous administration (%)	70.3
Time from renal biopsy to belimumab initiation (y)	1.2 [0.07–6.9]
Prescribed for renal involvement (%)	43.1
Laboratory baseline tests	
Creatinine (mg/dl)	0.9 (0.7)
eGFR (ml/min/1.73m^2^)	96.8 (30.4)
Albumin (g/dL)	3.8 (0.6)
Haemoglobin (g/dl)	12.3 (1.8)
Leukocytes (x10E3/microL)	6.5 (3.4)
Platelets (x10E3/microL)	245.3 (86.2)
Proteinuria (g/day)	0.58 [0.2-1.3]
Patients with >1g/day proteinuria (%)	33
Patients with >3.5 g/day proteinuria (%)	9
Anti-dsDNA (UI/ml)	170.5 (340.6)
C3 (mg/dl)	82.4 (32.4)
C4 (mg/dl)	15.1 (9.2)
CRP (mg/L)	6.6 (12.7)
ESR (mm/h)	36.2 (38.5)
Concomitant immunosuppression at belimumab initiation	
Patients receiving CS (%)	89.6
CS dose (mg)	10 [5–25]
Patients receiving MMF (%)	74.8
Patients receiving azathioprine (%)	7.9
Patients receiving cyclophosphamide (%)	3.0
Patients receiving CNI (%)	13.4
Patients receiving rituximab (%)	1.5
Patients receiving triple therapy (Belimumab+CS+3^rd^ drug; %)	80.7

Y, years; M, months; CRP, C Reactive Protein; ESR, Erythrocyte Sedimentation Rate; CS, corticosteroids; CNI, Calcineurin inhibitor; MMF, mycophenolate mophetil; eGFR, estimated Glomerular Filtration Rate; Anti-dsDNA, anti–double-stranded DNA antibodies.

Belimumab was administered in all cases according to the product’s prescribing information *(Benlysta SmPC* (26)*).* Renal involvement was the primary indication for treatment initiation (43.1%), with 27.6% of these patients starting therapy at LN onset. At belimumab initiation, 86.6% of patients were receiving hydroxychloroquine, 89.6% corticosteroids, and 80.7% combination therapy including a third agent, most commonly MMF. Median follow-up was 1.1 years [IQR 0.7–2.2], with a maximum follow-up of 12 years.

### Primary outcomes: PERR and CRR

At 6 months, 70.7% of patients achieved PERR, 71.1% at 12 months, and 72.7% at 24 months. CRR was reached by 52.4% of patients at 6 months, 47.6% at 12 months, and 49.1% at 24 months. Notably, at belimumab initiation, 54.9% of patients already met PERR criteria, and 39.9% met CRR criteria. After excluding these patients from the analysis, PERR was achieved by 56.3%, 57.8% and 61.9% of patients at 6, 12 and 24 months, respectively, whereas CRR was achieved by 40.9%, 37.7% and 46.2%, at the corresponding timepoints. The median time to achieve both PERR and CRR was 6 months IQR [6-6] (**Table 2**; **Supplementary Figure 2**).

**Table 2:** Primary outcomes and evolution of renal and serological parameters during follow-up with belimumab treatment.

Laboratory parameters	Baseline (n=202)	6 months (n=202)	12 months (n= 149)	24 months (n=59)
Serum Creatinine (mg/dl)	0.9 (0.7)	0.8 (0.3)	0.8 (0.3)	0.8 (0.3)
eGFR (ml/min/1.73m^2^)	96.8 (30.4)	98.3 (8.4)	95.6 (27.7)	98.4 (29.0)
Serum Albumin (g/dl)	3.8 (0.6)	4.0 (0.4)*	4.1 (0.5)*	4.12 (0.3)*
Proteinuria (g/day)	0.6 [0.21-1.35]	0.3 [0.1-0.6]*	0.3 [0.1-0.6]*	0.2[0.09-0.5]*
Hematuria (RBCs/HPF)	5 [0-20]	0.5 [0-6.5]*	1 [0-5]*	0.25 [0-5]*
Anti-dsDNA (UI/ml)	66.5 [23.5-182.8]	41 [16-93]*	34 [17-93]*	36 [13-105]*
C3 (mg/dl)	82.43 (32.4)	94.3 (25.9)*	94.8 (27.3)*	92.4 (21.5)*
C4 (mg/dl)	15.1 (9.2)	18.6 (8.8)*	20.3 (9.1)*	20.8 (8.5)*
ESR (mm/h)	36.2 (38.5)	19.5 (21.6)*	20.1 (21.1)*	15.0 (16.6)*
Outcomes				
PERR (n/N; %)	106/193 (54.9)	133/188 (70.7)*	133/188 (71.1)*	40/55 (72.7)
PERR (n/N; %) (baseline PERR = 0)	0	45/80 (56.3)*	37/64 (57.8)*	13/21 (61.9)*
CRR (n/N; %)	77/193 (39.9)	99/189 (52.4)#	68/143 (47.6)	27/55 (49.1)
CRR (n/N; %) (baseline CRR = 0)	0	45/65 (40.9)*	32/85 (37.7)*	12/26 (46.2)*

eGFR, Glomerular Filtration Rate; RBCs/HPF, red blood cells per high-power field; ESR, erythrocyte sedimentation rate; PERR, primary efficacy renal response; CRR, complete renal response; Anti-dsDNA, anti–double-stranded DNA antibodies. ^#^p<0.05; *p<0.001.

#### Comparison with BeRLiSS-LN and BLISS-LN cohorts

**Figure 1:**
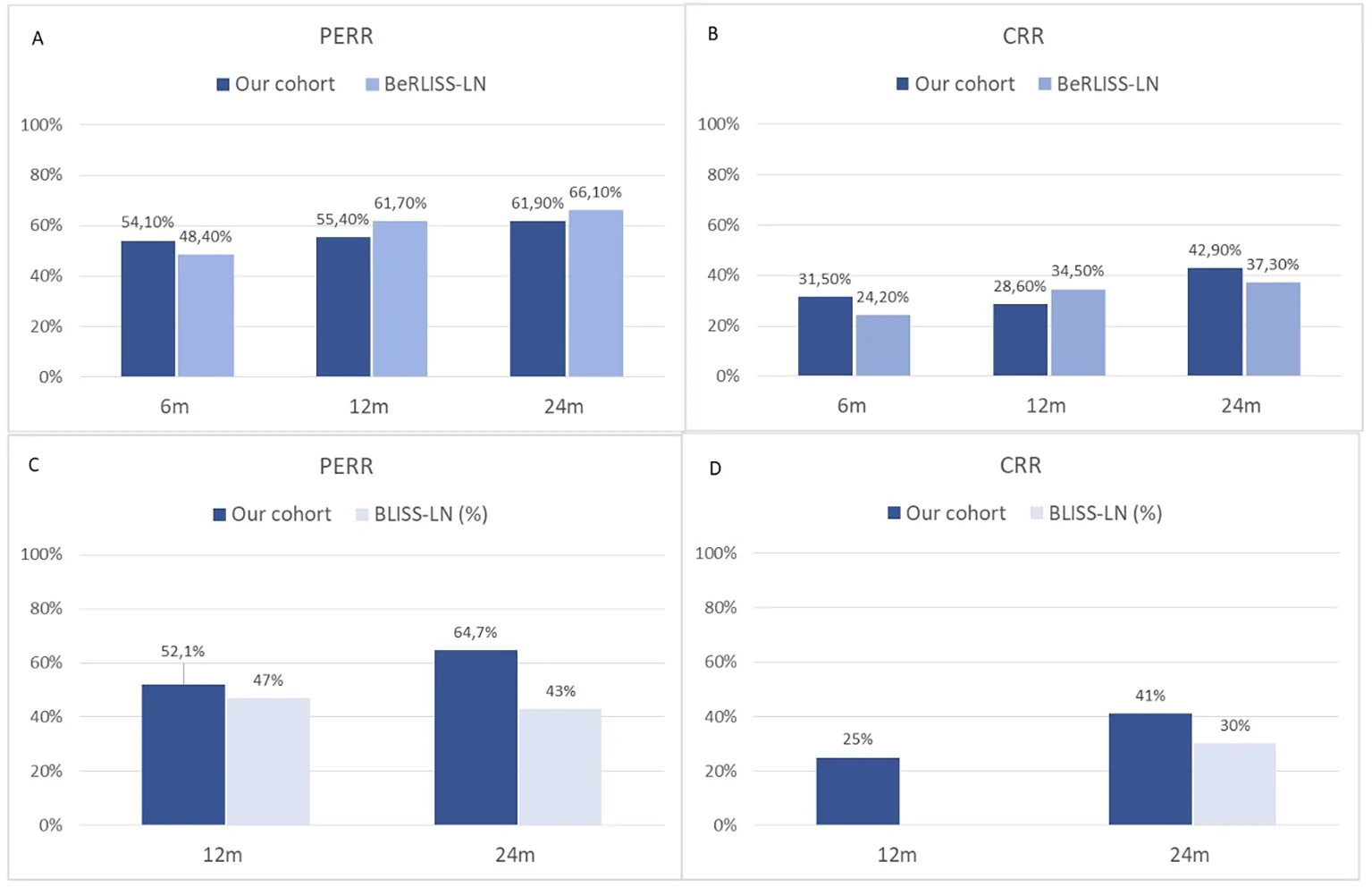
Comparison of the BeRLiSS-LN cohort with our subcohort meeting BeRLiSS-LN inclusion criteria **(A, B)**, and of the BLISS-LN cohort with our subcohort meeting BLISS-LN inclusion criteria **(C, D)**. PERR, primary efficacy renal response; CRR, Complete renal response.

- BeRLiSS-LN:
  At belimumab initiation, 153 patients (76% of our cohort) met inclusion criteria comparable to those of the BeRLiSS-LN study (18; **Supplementary Methods S1**). Based on BeRLiSS-LN outcome criteria, 61.9% of our subcohort achieved PERR and 42.9% achieved CRR at 24 months (**Figure 1**).
  *BeRLiSS-LN*: Applying BLISS-LN inclusion criteria (3), we selected patients with proteinuria ≥1 g/day at belimumab initiation (n = 68). Among these patients, 52.1% achieved PERR at 12 months. At 24 months, 64.7% achieved PERR and 41.2% CRR respectively (**Figure 1**).

However, these results should be interpreted with caution because of potential differences between our cohort and the BeRLiSS-LN and BLISS-LN populations with respect to baseline characteristics, disease activity concomitant treatment and study design.

### Secondary outcomes

A statistically significant improvement was observed in proteinuria, microhematuria, and serum albumin levels at 12 and 24 months, while renal function remained stable throughout follow-up (**Table 2**). Notably, compared with each patient´s baseline values, proteinuria decreased by 53.2% at 6 months (n=174; *p* < 0.001), 61.0% at 12 months (n=134; *p* < 0.001) and 59.2% at 24 months (n=52; *p* < 0.001) (**Supplementary Figure 3**).

**Figure 2:**
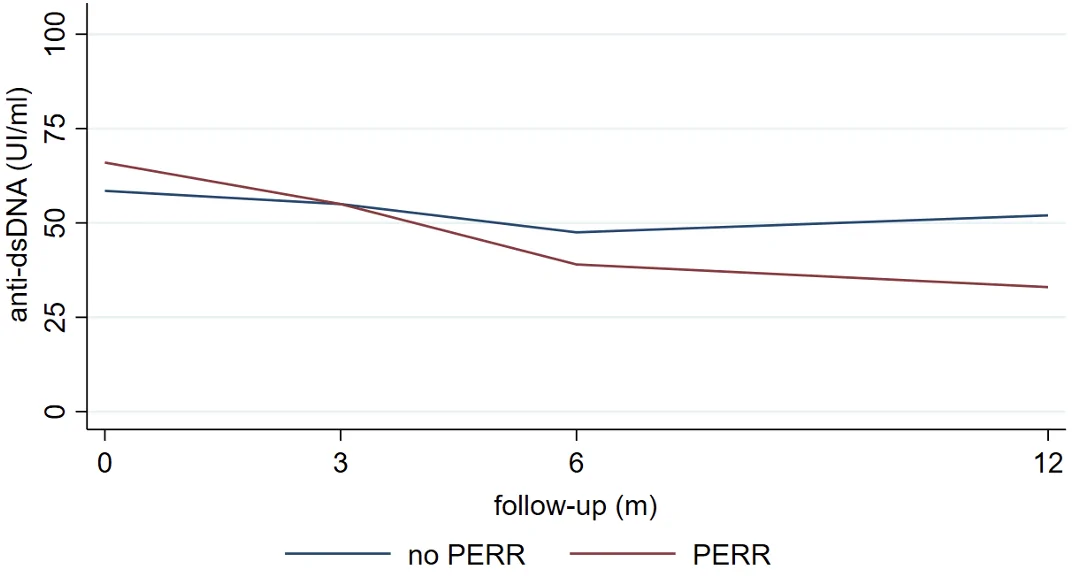
Serum anti-dsDNA levels during follow-up according to PERR status. PERR, primary efficacy renal response; Anti-dsDNA, anti–double-stranded DNA antibodies; m, months.

Significant improvements were also observed in immunological serologic markers of lupus activity, including anti-dsDNA antibodies, complement C3 and C4, and ESR, at 12 and 24 months of follow-up (**Supplementary Figure 4**). Notably, patients who achieved PERR exhibited greater reductions in anti-dsDNA levels than non-responders, although this did not reach statistical significance in the unadjusted analysis (*p* = 0.07) (**Figure 2**). The mean SLEDAI-2K score decreased from 14.5 (SD 9.0) at baseline to 4.1 (SD 6.3) at the end of follow-up (*p* < 0.001).

Among the 133 patients who achieved PERR at 6 months, only 2 (1%) experienced LN relapse based on laboratory criteria, although 12 (9.0%) required “prohibited medications” (**Supplementary Methods S1**). By the end of follow-up, only 2 patients required renal replacement therapy (RRT), and 1 patient died from non-renal causes (hemorrhagic shock).

### “Early” versus “late” initiation

**Figure 3:**
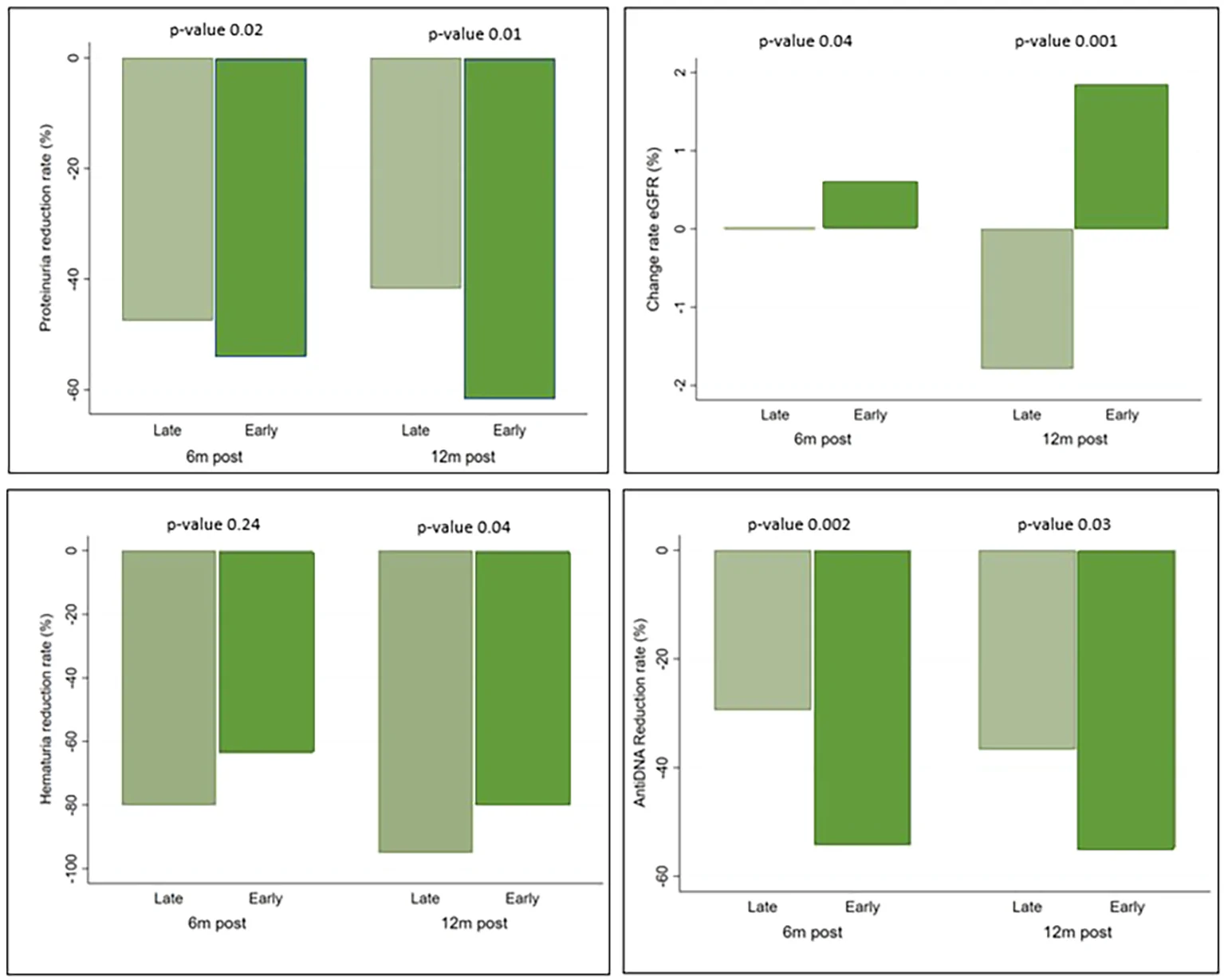
Changes in proteinuria, eGFR, hematuria and anti-dsDNA levels over time according to “early” versus “late” initiation of belimumab. m, months; eGFR, estimated glomerular filtration rate; anti-dsDNA, anti–double-stranded DNA antibodies.

The cohort was stratified by timing of belimumab initiation. Although the “early”-initiation group had worse baseline parameters, they demonstrated significantly greater improvements (Δ) in eGFR, proteinuria, microhematuria, anti-dsDNA antibodies, and SLEDAI-2K compared with the “late”-initiation group (**Table 3**; **Figure 3**; **Supplementary Table 1**).

**Table 3:** Comparison of renal and extrarenal parameters according to “early” versus “late” initiation of belimumab.

Renal and extrarrenal parameters	Baseline			12 months			Delta (Δ)		
	Early	Late	*p* value	Early	Late	*p* value	Early	Late	*p* value
N	39	146		39	146		39	146	
eGFR (ml/min/1.73m^2^)	95,22 (29,29)	96,21 (30,13)	0,86	100,99 (21,85)	92,52 (27,77)	0,137	+7.8 (19.5)	-1.8 (15.2)	0.006
Proteinuria (g/day)	2,27 (2,77)	1,03 (1,41)	<0.001	0,38 (0,31)	0,48 (0,67)	0.41	-1.6 (2.4)	-0.5 (1.0)	0.001
Anti-dsDNA (UI/ml)	68 [60–379]	56,5 [15–149]	0.008	38 [26–63]	29 [10–89]	0.15	-35.5 [-304 to -30]	-11.5 [-40 to 0]	0.005
SLEDAI 2K	17,83 (9,37)	13,46 (8,53)	0.008	1,54 (3,54)	4,08 (5,97)	0.01	-16.3 (8.5)	-9.2 (8.5)	<0.001
Hematuria (RBCs/HPF)	20 [5-20]	3 [0-11]	<0.001	4.5 [1-10]	0 [0-3]	<0.001	-11.5 [-40 to 0]	-1 [-10 to 0]	0.01

eGFR, Glomerular Filtration Rate; Anti-dsDNA, anti–double-stranded DNA antibodies, SLEDAI 2K, Systemic Lupus Erythematosus Disease Activity Index; RBCs/HPF, red blood cells per high-power field.

### Histological changes

Among patients who underwent a renal biopsy performed within 3 months before belimumab initiation, five patients had a protocol repeat biopsy after 3 years. The activity index improved in all cases [from 6 (5-10) to 1 (0-1)], whereas the chronicity index increased in four of the five patients [from 2 (1-3) to 5 (3-7)] (**Supplementary Figure 5**).

### Predictors of renal response

In multivariable logistic regression analysis, early initiation of belimumab emerged as an independent predictor of renal response, conferring an approximately fourfold increase in the odds of achieving response (OR 3.91, 95% CI 1.03–14.89; *p* = 0.045). Likewise, patients who achieved a ≥50% reduction in anti-dsDNA levels at 12 months had higher odds of achieving PERR (OR 3.91, 95% CI 1.00–15.30; *p* = 0.050) (**Supplementary Table 2**).

### Impact on concomitant immunosuppressive therapies

#### Corticosteroid reduction

At baseline, 89.6% of the overall cohort were receiving corticosteroids (median prednisone dose 10 mg/day [IQR 5–25]). Among these patients, those with ≥24 months of follow-up (n=54) were analyzed. A significant reduction in prednisone dose was observed as early as month 3 after initiation of belimumab (*p* < 0.001), decreasing to 5 mg/day [IQR 2.5–5.0] at 12 months and to 2.5 mg/day [IQR 0–5.0] at 24 months. Notably, 23.1% of patients were corticosteroid-free at 24 months. (**Supplementary Table 3**).

#### Reduction in mycophenolate mofetil

At belimumab initiation, 74.8% of the overall cohort were receiving MMF (median dose 2000 mg/day [IQR 1250–2000]). Among these patients, 45 had ≥24 months of follow-up. MMF dosage decreased progressively and significantly, from a median of 2000 mg/day [IQR 1500–2000] at baseline to 1000 mg/day [IQR 750–1500] at 24 months (*p* < 0.001; **Supplementary Table 3**).

### Adverse events and discontinuations

Overall, 9.9% of the cohort experienced a total of 21 medication-related adverse events, resulting in permanent discontinuation in only 6 patients (3.0%) (**Supplementary Table 4**). Additionally, Belimumab was discontinued in 17 patients for other reasons, including pregnancy planning or actual pregnancy in 4 cases, and lack of response in 11 cases.

## Discussion

We report the experience of a large Spanish cohort of patients with LN treated with belimumab in real-world clinical practice. Overall, we observed a high rate of renal response. Notably, earlier initiation of belimumab was associated with improved renal outcomes. Improvements were also observed in extrarenal disease parameters, and reductions in anti-dsDNA antibodies emerged as a predictor of renal response in our cohort. Furthermore, the rate of renal relapse was low, consistent with findings from clinical trials. Our findings further support the favorable safety profile of belimumab. Treatment was associated with reduced corticosteroid use, underscoring its steroid-sparing effect, and with decreased exposure to mycophenolate mofetil, without an increase in renal or extrarenal relapses. These findings warrant further investigation in future studies.

In this Spanish LN cohort, the PERR was 71.1% at 12 months and 72.7% at 24 months. Among patients who did not meet PERR criteria at the time of belimumab initiation, 57.8% and 61.9% achieved PERR at 12 and 24 months, respectively. These findings suggest that belimumab was associated with the maintenance or improvement of renal response in this real-world cohort of patients with LN. However, these findings should be interpreted with caution, as not all patients had reached 24 months of follow-up at database closure. Reported renal outcomes in LN vary considerably in the literature, largely owing to differences in baseline patient characteristics across cohorts and the lack of standardized outcomes definitions. To facilitate interpretation of our findings in the context of previous studies, we performed a subgroup analysis including only patients who fulfilled the inclusion criteria of the BLISS-LN and BeRLiSS-LN studies *(*3, 18*).* Among patients meeting the BeRLiSS-LN eligibility criteria, 61.9% achieved primary efficacy renal response (PERR) at 24 months, whereas 66.1% of patients achieved PERR at 24 months in the BeRLiSS-LN cohort (**Figure 1**). Similarly, among patients fulfilling the BLISS-LN inclusion criterion of baseline proteinuria ≥1 g/day, 52.1% achieved PERR at 12 months in our cohort, whereas the corresponding proportion was 47.0% in the BLISS-LN trial (**Figure 1**). However, these findings should be viewed in the context of differences between the study populations, including baseline characteristics, disease activity, concomitant treatments, and study design. In the absence of a control group, an intra-cohort analysis comparing proteinuria before and after belimumab initiation showed a statistically significant reduction, further supporting the efficacy of belimumab in this real-world setting.

Early initiation of belimumab in our cohort—within one month of histological diagnosis—was associated with significant improvements in renal outcomes, including reduced proteinuria, stabilized eGFR, and favorable extra-renal responses, reflected by declines in anti-dsDNA titers and SLEDAI-2K scores. In our cohort, early therapy remained an independent predictor of treatment response, underscoring the importance of intensive intervention before histopathological lesions progress to chronicity, a process shown to occur rapidly (27). Recent clinical guidelines increasingly recommend early triple therapy at treatment initiation for LN *(*15–17*)*, based on evidence from randomized controlled trials (3–5*)*. However, real-world data on the benefits of earlier initiation remain limited. Our findings align with the LOOPS registry and mechanistic studies demonstrating early reductions in double-negative B cells with belimumab, which correlate with improved renal outcomes *(*28–30*).* Collectively, these results support a paradigm in which prompt initiation of belimumab—particularly as part of an early, intensive combination regimen—may maximize therapeutic benefit in LN, consistent with current guideline recommendations.

We also observed improvement in serological immune parameters among our patients with LN. Following initiation of belimumab, serum levels of C3, C4, and anti-dsDNA significantly improved across the entire cohort, accompanied by reduction in SLEDAI-2K score. These findings are consistent with previous reports from cohorts of patients with both renal and non-renal SLE *(*3, 9, 14, 18, 19, 31, 32). The observed association between a ≥50% reduction in anti-dsDNA levels and PERR suggests that dynamic serological changes may better capture treatment response than single-time-point measurements. Even after adjustment for early treatment initiation, the directionality of the effect supports its potential utility as a biological marker of renal response. The lack of independent predictive value of C3 after adjustment could reflect shared variation with anti-dsDNA decline, supporting the intertwined nature of immune complex formation and complement consumption in SLE, although this remains still unclear. Although the role of anti-dsDNA remains debated, increasing evidence supports its relevance as a biomarker of SLE disease activity, particularly when evaluated longitudinally rather than as a single absolute measurement (33). This is further supported by anti-dsDNA seroconversion observed in patients treated with chimeric antigen receptor T-cell therapy, highlighting its dynamic relationship with disease activity *(*34*).* In LN specifically, anti-dsDNA appears to have prognostic relevance, particularly in proliferative classes (35), consistent with our cohort, which comprised 79.7% proliferative LN cases (class III or IV) and 8.9% mixed classes (III/IV plus V).

From a histological perspective, although our subgroup was very small to permit definitive conclusions, our findings align with those reported by (36), showing a significant improvement in the activity index (AI) among patients treated with belimumab. Consistent with previous reports, we also observed worsening of the chronicity index (CI), likely reflecting the inherent impact of each disease flare (31). These data underscore that minimizing renal flares remains the most effective strategy to improve long-term renal prognosis. Notably, our cohort exhibited a low rate of renal relapse, comparable to that reported in the *post-hoc* analysis of the pivotal trial (14.4% vs 23% in the placebo arm; corresponding to a 55% relative risk reduction in renal flares; hazard ratio 0.45 [95% CI 0.28–0.72]; *p* < 0.0008) (12), suggesting that belimumab may help maintain LN remission. Furthermore, the favorable safety profile of belimumab, together with its corticosteroid-sparing effect, consistently demonstrated across studies and confirmed in our cohort, supports its long-term utility *(*3, 8–11, 14, 32*)*. We also observed a significant reduction in MMF exposure during follow-up, from a median of 2000 mg/day [IQR 1500–2000] at baseline to 1000 mg/day [IQR 750–1500] at 24 months (*p* < 0.001). Although this finding requires confirmation in prospective studies, it further highlights the potential of belimumab as a valuable therapeutic option for the medium- and long-term management of LN.

Our study has several strengths. It provides further real-world evidence that belimumab use is associated with maintenance or improvement of renal outcomes in patients with LN, consistent with previous reports, and adds novel evidence supporting the prognostic benefit of early drug initiation. It also highlights the potential utility of changes in anti-dsDNA levels as a biomarker of renal response in proliferative LN. Progressive reductions in corticosteroid and mycophenolate mofetil doses, a low rate of renal flares, and the favorable safety profile of belimumab further support its role in long-term disease management. Limitations include the retrospective design, the absence of a comparative control cohort, variability in the timing and conditions of belimumab initiation across patients, the progressively decreasing number of evaluable patients at later follow-up time points, which may have introduced attrition bias and may have led to an overestimation of treatment response, and the small number of repeat biopsies, which precluded definitive conclusions regarding the histological findings.

In conclusion, in this real-world cohort, belimumab was associated with the achievement or maintenance of renal response in patients with LN. Early initiation, coupled with reductions in corticosteroid and mycophenolate mofetil use, a favorable safety profile, and a low incidence of renal flares, further supports its potential role in both early intervention and log-term management of LN. Notably, a ≥50% reduction in anti-dsDNA antibody titers during follow-up was independently associated with primary renal response, suggesting that dynamic changes in anti-dsDNA antibody titers may represent a practical prognostic biomarker in patients with predominantly proliferative LN treated with belimumab. Prospective, controlled studies with extended follow-up are needed to confirm these findings.

## Data Availability

The raw data supporting the conclusions of this article will be made available by the authors, without undue reservation.

## References

[1] Almaani S Meara A Rovin BH . Update on lupus nephritis. Clin J Am Soc Nephrol. (2017) 12:825. doi: 10.2215/cjn.05780616 27821390 PMC5477208

[2] Cervera R Khamashta MA Font J Sebastiani GD Antonio Gil Lavilla P . Morbidity and mortality in systemic lupus erythematosus during a 10-year period: a comparison of early and late manifestations in a cohort of 1,000 patients. Medicine. (2003) 82:299–308. doi: 10.1097/01.md.0000091181.93122.55 14530779

[3] Furie R Rovin BH Houssiau F Malvar A Teng YKO Contreras G . Two-year, randomized, controlled trial of belimumab in lupus nephritis. N Engl J Med. (2020) 383:1117–28. doi: 10.1056/nejmoa2001180 32937045

[4] Saxena A Ginzler EM Gibson K Satirapoj B Santillán AEZ Levchenko O . Safety and efficacy of long-term voclosporin treatment for lupus nephritis in the phase 3 AURORA 2 clinical trial. Arthritis Rheumatol. (2024) 76:59–67. doi: 10.1002/art.42657 37466424

[5] Furie R Rovin BH Garg JP Santiago MB Aroca-Martínez G Zuta Santillán AE . Efficacy and safety of obinutuzumab in active lupus nephritis. N Engl J Med. (2025) 392:1471–83. doi: 10.1056/nejmoa2410965 39927615

[6] Baker KP Edwards B Main SH Choi GH Wager RE Halpern WG . Generation and characterization of LymphoStat-B, a human monoclonal antibody that antagonizes the bioactivities of B lymphocyte stimulator. Arthritis Rheum. (2003) 48:3253–65. doi: 10.1002/art.11299 14613291

[7] Dennis GJ . Benlysta: a BLyS-specific inhibitor for the treatment of systemic lupus erythematosus. Clin Pharmacol Ther. (2012) 91:143–9. doi: 10.1038/clpt.2011.290 22130121

[8] Navarra SV Guzmán RM Gallacher AE Hall S Levy RA Jimenez RE . Efficacy and safety of belimumab in patients with active systemic lupus erythematosus: a randomised, placebo-controlled, phase 3 trial. Lancet. (2011) 377:721–31. doi: 10.1016/s0140-6736(10)61354-2 21296403

[9] Furie R Petri M Zamani O Cervera R Wallace DJ Tegzová D . A phase III, randomized, placebo-controlled study of belimumab, a monoclonal antibody that inhibits B lymphocyte stimulator, in patients with systemic lupus erythematosus. Arthritis Rheum. (2011) 63:3918–30. doi: 10.1002/art.30613 22127708 PMC5007058

[10] Brunner HI Abud-Mendoza C Viola DO Calvo Penades I Levy D Anton J . Safety and efficacy of intravenous belimumab in children with systemic lupus erythematosus: results from a randomised, placebo-controlled trial. Ann Rheum Dis. (2020) 79:1340–8. doi: 10.1136/annrheumdis-2020-217101 32699034 PMC7509523

[11] Furie R Rovin BH Houssiau F Contreras G Teng YKO Curtis P . Safety and efficacy of belimumab in patients with lupus nephritis: open-label extension of BLISS-LN study. Clin J Am Soc Nephrol. (2022) 17:1620–30. doi: 10.2215/cjn.02520322 36302567 PMC9718049

[12] Rovin BH Furie R Teng YKO Contreras G Malvar A Yu X . A secondary analysis of the Belimumab International Study in Lupus Nephritis trial examined effects of belimumab on kidney outcomes and preservation of kidney function in patients with lupus nephritis. Kidney Int. (2022) 101:403–13. doi: 10.1016/j.kint.2021.08.027 34560137

[13] Anders HJ Furie R Malvar A Zhao MH Hiromura K Weinmann-Menke J . Effect of belimumab on kidney-related outcomes in patients with lupus nephritis: post hoc subgroup analyses of the phase 3 BLISS-LN trial. Nephrol Dial Transplant. (2023) 38:2733–42. doi: 10.1093/ndt/gfad167 37463054 PMC10689192

[14] Stohl W Schwarting A Okada M Scheinberg M Doria A Hammer AE . Efficacy and safety of subcutaneous belimumab in systemic lupus erythematosus: a fifty-two-week randomized, double-blind, placebo-controlled study. Arthritis Rheum. (2017) 69:1016–27. doi: 10.1002/art.40049 PMC5434872 28118533

[15] Kidney Disease: Improving Global Outcomes (KDIGO) Glomerular Diseases Work Group . KDIGO 2024 clinical practice guideline for the management of glomerular diseases. Kidney Int. (2024) 105:S1–S69. doi: 10.1016/j.kint.2023.09.002 38182286

[16] Sammaritano LR Askanase A Bermas BL Dall'Era M Duarte-García A Hiraki LT . 2024 American College of Rheumatology (ACR) guideline for the screening, treatment, and management of lupus nephritis. Arthritis Rheumatol. (2025) 77(9):1115–35. doi: 10.1002/art.43212 40331662

[17] Fanouriakis A Kostopoulou M Anders HJ Andersen J Aringer M Beresford MW . EULAR recommendations for the management of systemic lupus erythematosus with kidney involvement: 2025 update. Ann Rheum Dis. (2026) 85:75–90. doi: 10.1016/j.ard.2025.09.007 41107121

[18] Gatto M Saccon F Andreoli L Bartoloni E Benvenuti F Bortoluzzi A . Durable renal response and safety with add-on belimumab in patients with lupus nephritis in real-life setting (BeRLiSS-LN). J Autoimmun. (2021) 124:102729. doi: 10.1016/j.jaut.2021.102729 34600347

[19] Lin S Zhang J You X Chen B Liang Y Zhou Y . Efficacy and safety of belimumab in patients with lupus nephritis: a real-world retrospective observational study. Rheumatol (Oxford). (2025) 64:614–22. doi: 10.1093/rheumatology/kead707 38145498

[20] Ma Y Li X Yu X Lan P Liang Y Lu W . Efficacy and safety of belimumab in refractory and newly diagnosed active lupus nephritis patients: a real-world observational study. Clin Kidney J. (2025) 18:sfaf103. doi: 10.1093/ckj/sfaf103 40416394 PMC12100164

[21] Tan M Xu J Tan Y Qu Z Yu F Zhao M . Efficacy and safety of belimumab in lupus nephritis patients: a real-world observational study in China. Kidney Dis. (Basel). (2023) 9:218–28. doi: 10.1159/000529675 PMC10368010 37497208

[22] Sakai H Miyazaki Y Nakayamada S Kubo S Hanami K Fukuyo S . Efficacy, safety and optimal intervention of belimumab for proliferative lupus nephritis patients in real-world settings: LOOPS registry. Rheumatol. (Oxford). (2025) 64:1930–9. doi: 10.1093/rheumatology/keae495 39288317

[23] Aringer M Costenbader K Daikh D Brinks R Mosca M Ramsey-Goldman R . European League Against Rheumatism/American College of Rheumatology classification criteria for systemic lupus erythematosus. Ann Rheum Dis. (2019) 78:1151–9. doi: 10.1136/annrheumdis-2018-214819 31383717

[24] Bajema IM Wilhelmus S Alpers CE Bruijn JA Colvin RB Cook HT . Revision of the International Society of Nephrology/Renal Pathology Society classification for lupus nephritis: clarification of definitions, and modified National Institutes of Health activity and chronicity indices. Kidney Int. (2018) 93:789–96. doi: 10.1016/j.kint.2017.11.023 29459092

[25] Zonozi R Aqeel F Le D Cortazar FB Thaker J Zabala Ramirez MJ . Real-world experience with Avacopan in antineutrophil cytoplasmic autoantibody-associated vasculitis. Kidney Int Rep. (2024) 9:1783–91. doi: 10.1016/j.ekir.2024.03.022 38899183 PMC11184253

[26] European Medicines Agency . Benlysta (Belimumab): Summary of Product Characteristics (Smpc) (2026). Available online at: https://www.ema.europa.eu/en/documents/product-information/benlysta-epar-product-information_en.pdf (Accessed April 14, 2026).

[27] Malvar A Alberton V Lococo B Lourenco M Martinez J Burna L . Remission of lupus nephritis: the trajectory of histological response in successfully treated patients. Lupus Sci Med. (2023) 10:e000932. doi: 10.1136/lupus-2023-000932 37258036 PMC10255076

[28] Sakai H Miyazaki Y Nakayamada S Kubo S Hanami K Fukuyo S . Efficacy, safety and optimal intervention of belimumab for proliferative lupus nephritis patients in real-world settings: LOOPS registry. Rheumatol (Oxford). (2025) 64:1930–9. doi: 10.1093/rheumatology/keae495 39288317

[29] Jacobi AM Huang W Wang T Freimuth W Sanz I Furie R . Effect of long-term belimumab treatment on B cells in systemic lupus erythematosus: extension of a phase II, double-blind, placebo-controlled, dose-ranging study. Arthritis Rheum. (2010) 62:201–10. doi: 10.1002/art.27189 20039404 PMC2857977

[30] You X Zhang R Shao M He J Chen J Liu J . Double negative B cell is associated with renal impairment in systemic lupus erythematosus and acts as a marker for nephritis remission. Front. Med. (Lausanne). (2020) 7:85. doi: 10.3389/fmed.2020.00085 32318574 PMC7155774

[31] Stohl W Hiepe F Latinis KM Thomas M Scheinberg MA Clarke A . Belimumab reduces autoantibodies, normalizes low complement levels, and reduces select B cell populations in patients with systemic lupus erythematosus. Arthritis Rheum. (2012) 64:2328–37. doi: 10.1002/art.34400 22275291 PMC3350827

[32] Collins CE Cortes-Hernández J Garcia MA von Kempis J Schwarting A Touma Z . Real-world effectiveness of belimumab in the treatment of systemic lupus erythematosus: pooled analysis of multi-country data from the OBSErve studies. Rheumatol Ther. (2020) 7:949–65. doi: 10.1007/s40744-020-00243-2 33206344 PMC7695800

[33] Yeo AL Kandane-Rathnayake R Koelmeyer R Golder V Louthrenoo W Chen YH . SMART-SLE: serology monitoring and repeat testing in systemic lupus erythematosus—an analysis of anti-double-stranded DNA monitoring. Rheumatol. (Oxford). (2024) 63:525–33. doi: 10.1093/rheumatology/kead231 PMC10836977 37208196

[34] Mackensen A Muller F Mougiakakos D Böltz S Wilhelm A Aigner M . Anti-CD19 CAR T cell therapy for refractory systemic lupus erythematosus. Nat Med. (2022) 28:2124–32. doi: 10.1038/s41591-022-02017-5 36109639

[35] Fava A Wagner CA Guthridge CJ Kheir J Macwana S DeJager W . Association of autoantibody concentrations and trajectories with lupus nephritis histologic features and treatment response. Arthritis Rheumatol. (2024) 76:1611–22. doi: 10.1002/art.42941 38962936 PMC11521769

[36] Malvar A Alberton V Recalde C Heguilen R . Repeat kidney biopsy findings of lupus nephritis patients in clinical remission treated with mycophenolate associated with belimumab or mycophenolate plus standard of care therapy. Lupus. (2023) 32:1394–401. doi: 10.1681/asn.20223311s1185a 37754750

