# Supplementary material for: Real-world experience with belimumab in lupus nephritis: insights from the Spanish cohort
Source: Front Immunol. 2026 Aug 25;17:1897450. doi: 10.3389/fimmu.2026.1897450 (PMC13550196; doi:10.3389/fimmu.2026.1897450)
Supplement: Supplementary file 1 [file Supplementaryfile1.docx]

**SUPPLEMENTARY MATERIAL:**

**SUPPLEMENTARY METHODS S1:**

- ***Definition of Treatment Failure (use of rescue therapy):***

Treatment failure was defined according to the BLISS-LN trial criteria. Patients were required to taper glucocorticoids to ≤10 mg/day by week 24 and to not exceed this dose through week 104, except for protocol-allowed short-term rescue treatment between weeks 24 and 76 for reasons other than lupus nephritis. No glucocorticoid rescue treatments were allowed between weeks 76 and 104. Patients were considered to have treatment failure if they violated the glucocorticoid rules or received additional immunosuppressive agents (except topical agents) beyond the induction and maintenance regimens; initiated the use of Angiotensin-converting enzyme (ACE) inhibitors, Angiotensin II Receptor Blockers (ARBs) or antimalarial drugs after week 24; or if the standard therapy (cyclophosphamide, azathioprine or mycophenolate mofetil) exceeded permitted doses.^1^

- ***Comparison with the BeRLiSS-LN cohort:***

To enable comparison between our cohort and the BeRLiSS-LN study cohort, we first selected patients from our cohort with “renal activity” according to criteria similar to those used in BeRLiSS-LN: proteinuria ≥ 0.5 g/day and/or active urinary sediment defined as ≥5 red blood cells per high-power field, and/or estimated glomerular filtration rate (eGFR) ≤60 mL/min/1.73 m²; accompanied by positive anti-dsDNA antibodies and/or low C3/C4 levels when these data were available. Renal response outcomes were assessed according to the definitions adopted in the BeRLiSS-LN study. “Primary efficacy renal response” (PERR) was defined as proteinuria ≤0.7 g/24 h, eGFR ≥60 mL/min/1.73 m², and no use of rescue therapy. “Complete renal response” (CRR) was defined as proteinuria <0.5 g/24 h, eGFR ≥90 mL/min/1.73 m², and no use of rescue therapy.^2^

- ***Lupus nephritis (LN) flare:***

For LN flare, we used the same definition as in the post hoc analysis of the BLISS-LN trial: (i) impaired kidney function (i.e. reproducible eGFR decrease of >20% from week 24) accompanied by proteinuria (uPCR >1 g/g) and/or cellular casts (red blood cells. white blood cells in the absence of infection. or both). or (ii) an increase in proteinuria compared to week 24. or (iii) treatment failure due to kidney disease–related intake of prohibited medications^.^ An increase in proteinuria was defined as an increase in uPCR of >1 if week 24 uPCR was <0.2 g/g. uPCR >2.0 g/g if week 24 uPCR was 0.2 to 1 g/g. or as a doubling of uPCR if week 24 uPCR was >1 g/g.^3^

**SUPPLEMENTARY REFERENCES:**

1. Furie R, Rovin BH, Houssiau F, Malvar A, Teng YKO, Contreras G, et al. Two-Year, Randomized, Controlled Trial of Belimumab in Lupus Nephritis. N Engl J Med. 2020 Sep 17;383(12):1117-1128.
2. Gatto M, Saccon F, Andreoli L, Bartoloni E, Benvenuti F, Bortoluzzi A, et al. (2021) Durable renal response and safety with add-on belimumab in patients with lupus nephritis in real-life setting (BeRLiSS-LN). *J Autoimmun.* 124:102729.
3. Rovin BH, Furie R, Teng YKO, Contreras G, Malvar A, Yu X, et al. A secondary analysis of the Belimumab International Study in Lupus Nephritis trial examined effects of belimumab on kidney outcomes and preservation of kidney function in patients with lupus nephritis. Kidney Int. 2022 Feb;101(2):403-413.

**SUPPLEMENTARY TABLES:**

 Supplementary Table S1: Baseline data according group

|  | Late | Early | p-value |
| --- | --- | --- | --- |
| N | 146 | 39 | 0.2 |
| Age (y) | 37.9 (13.3) | 35.2 (11.2) | 0.9 |
| Male (%) | 12.3 | 13.2 |  |
| Ethnicity (%) |  |  | 0.9 |
| Caucasian | 71.2 | 71.8 |  |
| African American | 3.4 | 2.6 |  |
| Asian | 2.7 | 5.1 |  |
| Hispanic | 22.6 | 20.5 |  |
| Hypertension (%) | 39.7 | 15.4 | 0.005 |
| Diabetes Mellitus (%) | 2.8 | 2.6 | 0.9 |
| Patients with previous relapses (%) | 60.3 | 30.8 | <0.001 |
| Follow-up (y) | 2.0 (1.7) | 1.7 (0.9) | 0.4 |
| **Histology** |  |  | 0.09 |
| Classes III or IV (%) | 78.8 | 87.2 |  |
| Class V (%) | 13.0 | 0 |  |
| Mixed class (%) | 7.5 | 10.3 |  |
| Podocytopathy (%) | 0.7 | 2.6 |  |
| Activity index | 6.9 (4.7) | 9.4 (3.8) | 0.08 |
| Chronicity index | 1.9 (2.7) | 1.3(1.2) | 0.2 |
| Globally sclerosed glomeruli (%) | 6.3 (15.6) | 2.3 (5.0) | 0.01 |
| Crescents (%) | 4.9 (13.8) | 4.2 (14.8) | 0.8 |
| Interstitial fibrosis (%) | 8.9 (12.5) | 12.5 (12.8) | 0.12 |
| **Belimumab initiation** |  |  |  |
| Subcutaneous administration (%) | 71.9 | 82.1 | 0.2 |
| Time from renal biopsy to belimumab initiation (y) | 6.0 (6.8) | 0.02 (0.03) | <0.001 |
| Prescribed for renal involvement (%) | 39.0 | 66.7 | 0.002 |
| **Concomitant immunosuppression at belimumab initiation** |  |  |  |
| Patients receiving CS (%) | 85.6 | 100 | 0.01 |
| Dosis CS (mg) | 7.5 [5-20] | 25 [25-30] | <0.001 |

 Supplementary Table S2: Logistic regression analysis to identify predictors of primary efficacy renal response (PERR)

|  | **UNIVARIANT** | **MULTIVARIANT** |
| --- | --- | --- |
|  | **OR [CI 95%]** | **OR [CI 95%]** |
| **Baseline anti-dsDNA >15 UI/ml** | 1.77 [0.72-4.40] |  |
| **C3 < 90 mg/dl** | 3.43 [1.36-8.70] |  |
| **C4 > 10 mg/dl** | 3.81 [1.27-11.40] |  |
| **previous eGFR > 60 ml/min/1.73m^2^** | 1.00 [0.98-1.01] |  |
| **Proteinuria > 1 g/day** | 0.32 [0.10-1.03] |  |
| **Treatment due to renal involvement** | 0.64 [0.23-1.73] |  |
| **Number of previous relapses** | 0.83 [0.62-1.11] |  |
| **Anti-dsDNA reduction**  **(ref: no reduction)** | 1 | 1 |
| <50% Reduction of anti-dsDNA at 12m vs no reduction | 1.31 [0.39-4.34] | 1.29 [0.34-4.96] |
| ≥ 50% Reduction of anti-dsDNA  at 12m vs no or 0-50% reduction | 3.12 [0.93-10.46] | 3.91 [1.0-15.3] |
| <25% Reduction of anti-dsDNA at 6m from baseline (ref) | 1 |  |
| ≥ 25% Reduction of anti-dsDNA at 6m | 2.50 [0.96-6.50] |  |
| **Early vs late initiation** | 2.4 [0.8-6.8] | 3.91 [1.03-14.9] |
| **C3_reduction** | 1 |  |
| c3_increase 0-25 | 1.03 [0.28-3.75 |  |
| c3_increase>25 | 1.28 [0.40-4.07] |  |
| **Urinary sediment**  **> 5 RBCs/HPF** | 1.44 [0.59-3.55] |  |
| **Urinary sediment**  **>5 WBCs/HPF** | 2.49 [0.93-6.67] |  |
| **Crescents (%) in renal biopsy** | 0.99 [0.97-1.02] |  |

*Anti-dsDNA: anti–double-stranded DNA antibodies; eGFR: estimated glomerular filtration rate; RBCs/HPF: red blood cells per high-power field; WBCs/HPF white blood cells per high-power field; m: months; OR: odd ratio; CI: confidence interval*

Supplementary Table S3: Pattern of corticosteroids and MMF use following initiation of belimumab therapy

|  | Baseline | 3 months | 6 months | 12 months | 24 months |
| --- | --- | --- | --- | --- | --- |
| Patients on Corticosteroids (%) | 100 | 100 | 96.2 | 86.8 | 76.9 |
| Corticosteroids (mg) | 11.3 [5.0-30.0] | 6.8 [5.0-10.0]* | 5.0 [2.5-7.5]* | 5.0 [2.5-5.0]* | 2.5 [0-5.0]* |
|  | **Baseline** | **3 months** | **6 months** | **12 months** | **24 months** |
| Patients on MMF (%) | 100 | 100 | 95.5 | 91.1 | 85.7 |
| MMF (mg) | 2000 [1500-2000] | 2000 [1500-2000] | 2000 [1250-2000] | 2000 [1000-2000]# | 1000 [750-1500]* |
| *MMF: mycophenolate mophetil; *: p<0.001;* #*: p<0.05.* | | | | | |

| Adverse Event | Total of patients | Patients with permanent discontinuation of the drug |
| --- | --- | --- |
|  |  |  |
| ●     Infectious |  |  |
| Respiratory infection | 2 | 1 |
| Urinary tract infection | 2 | 1 |
| ●     Non-infectious |  |  |
| Skin rash | 1* | 1* |
| Pruritus | 2 | 0 |
| Facial flushing | 1 | 0 |
| Bronchospasm | 1 | 0 |
| Headache | 3 | 1 |
| Emotional lability or depression | 3 | 1 |
| Diarrhea | 2 | 0 |
| Leukopenia | 1 | 1 |
| Asthenia | 3* | 1* |
| *(*same patient)* |  |  |

Supplementary Table S4: Adverse Events

**SUPPLEMENTARY FIGURES:**

Supplementary Figure S1: Patient Selection Flowchart

Supplementary Figure S2: Renal response curve according to the Kaplan-Meier model, bases on BLISS-LN criteria; A) Principal efficacy renal response; B) Complete renal response

Supplementary Figure S3: Within-patient proteinuria changes during follow-up

p-value < 0.001

p-value 0.001

*Figure legend: m: months*

Supplementary Figure S4: – Serum C3, C4, and anti-dsDNA levels during follow-up

*Figure legend: Anti-dsDNA: anti–double-stranded DNA antibodies.*

*.*

Supplementary Figure S5: Changes in activity and chronicity indices in re-biopsied patients

*Figure legend: pat: patient.*
